# Effects of deceleration profiles and driving speed on usability and user perception in autonomous wheelchairs

**DOI:** 10.3389/fpsyg.2026.1850976

**Published:** 2026-06-30

**Authors:** Yihun Jeong, Joon Ho Lee, Sun Ho Lee, Dahae Park, Nahyeon Hwang, Woo Young Choi, Dongwook Hwang

**Affiliations:** 1 Department of Industrial Engineering, Keimyung University, Daegu, Republic of Korea; 2 Department of Intelligent Robot Engineering, Pukyong National University, Busan, Republic of Korea; 3 Strategic Planning Division, R&D Department, AI&ICT Convergence Team, Korea Intelligent Automotive Parts Promotion Institute, Daegu, Republic of Korea; 4 Department of Control and Instrumentation Engineering, Pukyong National University, Busan, Republic of Korea; 5 School of Media and Communication, Kwangwoon University, Seoul, Republic of Korea

**Keywords:** autonomous wheelchair, deceleration profile, driving speed, personal mobility, user perception

## Abstract

Autonomous wheelchairs are increasingly recognized as personal mobility systems that may serve both individuals with physical impairments and able-bodied users; however, little is known about how specific motion control parameters shape user experience during autonomous navigation. This study investigated the effects of deceleration profiles and driving speed on the usability of an autonomous wheelchair, along with changes in user perceptions following actual use. Twenty participants evaluated five deceleration modes (ranging from −0.2 m/s^2^ to −1.0 m/s^2^) and five driving speed modes (ranging from 3 km/h to 7 km/h) on five usability measures (safety, rapidity, accuracy, convenience, and preference), and their perceptions of the autonomous wheelchair were compared before and after riding the system. The results indicated that moderate deceleration profiles and an intermediate driving speed received the highest usability ratings, whereas both extreme deceleration and high speed were rated least favorably. Furthermore, trust-related perceptions, including intention to use, reliability, and safety, significantly improved after riding the autonomous wheelchair. The results provide empirical design guidelines for the human-centered development of autonomous personal mobility systems.

## 1 Introduction

Autonomous wheelchairs prioritize safety and comfort, as they are designed to support users with physical impairments and reduced postural stability. For these users, even minor motion disturbances can directly affect physical safety and psychological well-being (Rushton et al., 2015; Wang et al., 2013). Accordingly, wheelchair design has traditionally emphasized conservative motion control strategies to minimize discomfort, instability, and risk during everyday travel (Kirby et al., 1996).

With the introduction of autonomy, however, the role of the wheelchair extends beyond that of traditional wheelchairs. By enabling independent navigation without continuous caregiver assistance and reducing the need for manual control, autonomous wheelchairs are increasingly discussed as personal mobility systems that support not only short-distance travel in public and shared environments, but also users' social participation and engagement in everyday activities (Rushton et al., 2017). Importantly, the safety- and comfort-oriented design principles originally developed for users with impairments also make autonomous wheelchairs potentially valuable for able-bodied users, particularly in contexts where stable, predictable, and low-speed mobility is desired. Thus, autonomous wheelchairs have the potential to serve as personal mobility systems for able-bodied users as well.

Because autonomous personal mobility systems operate in dynamic public spaces and must continuously perceive and respond to their surroundings, they require precise motion planning and reliable control. In this context, deceleration is a key motion dynamic shaping safety, comfort, and overall user experience in autonomous wheelchair navigation. Previous studies have examined the effects of motion dynamics on passenger experience, establishing a clear link between braking profiles and physical stability.

In the broader context of transportation, (Abernethy et al. 1980) identified practical deceleration thresholds in public transit beyond which even seated, unrestrained passengers lose postural stability, demonstrating that excessive braking poses direct physical safety risks. At the same time, overly cautious or slow deceleration may preserve ride smoothness but can compromise safety if the wheelchair fails to stop in time to avoid collisions. Although gradual deceleration has been shown to reduce instability, the system must still respond effectively to hazards in dynamic environments. Prior work in transportation ergonomics further suggests that there is no single deceleration threshold that is comfortable for all users, due to individual differences in physical capability and perception (Hoberock, 1976). As a result, designers must aim for deceleration ranges that feel intuitively safe and controllable to most users, rather than optimizing solely for mechanical limits.

Beyond physical stability, braking characteristics also influence users' psychological experience during autonomous travel. Research indicates that smooth and predictable braking is closely associated with positive user evaluations. (Detjen et al. 2021) showed that erratic movements or harsh braking can quickly undermine user confidence, whereas smooth navigation supports a sense of safety. Similarly, (Koglbauer et al. 2018) showed that perceived trust and safety in an autonomous braking system decrease when the braking strategy is perceived as inappropriate or poorly adapted to the driving context. Also, (de Winkel et al. 2023) demonstrated that proactive and gentle deceleration in response to obstacles improves subjective comfort. Together, these studies suggest that braking serves not only a mechanical function but also shapes how users interpret system behavior during interaction.

In addition to braking dynamics, existing literature highlights driving speed as a key factor influencing comfort and perceived safety in autonomous wheelchairs. Higher speeds have been associated with increased anxiety and reduced perceived control, whereas very low speeds may be experienced as inefficient or frustrating (Petermeijer et al., 2017). Studies of wheelchair-seated passengers further suggest that overall ride conditions, including speed-related factors, contribute to perceived comfort and safety (Wretstrand et al., 2009). Taken together, these findings indicate that both driving speed and deceleration are critical design parameters in autonomous personal mobility systems, each requiring careful calibration to support a positive user experience.

Despite these insights, important knowledge gaps remain regarding what specific values of driving speed and deceleration best support user experience in autonomous wheelchairs. Although prior work suggests that both parameters matter, appropriate design ranges for each have not been systematically established, particularly when autonomous wheelchairs are considered as personal mobility systems for broader user groups. Identifying suitable values for these parameters is essential, as they influence users' perceptions of safety, comfort, and overall usability of autonomous wheelchair systems.

To address these knowledge gaps, this study aimed to identify suitable deceleration profiles and driving speed values that support positive user experience in autonomous wheelchairs. In this study, autonomous wheelchairs were conceptualized as personal mobility systems that may also be used by able-bodied individuals. Five core usability outcomes were considered: safety, rapidity, accuracy, convenience, and preference. Multiple braking profiles and driving speeds were implemented as experimental conditions to compare their effects and determine appropriate design ranges for each parameter. Through this approach, the study sought to inform the human-centered design of autonomous personal mobility systems that balance effective navigation with user experience.

## 2 Methods

### 2.1 Participants

Twenty individuals (10 males and 10 females) participated in this study. All participants were in their 20s (mean age: 23.25 years, SD: 0.89) and had normal or corrected-to-normal vision in both eyes. None of the participants indicated having any current musculoskeletal disorders. Prior to participation, each participant was fully informed of the study's purpose and procedures and signed an informed consent form. The research protocol was reviewed and approved by the Institutional Review Board of Pukyong National University (Approval No. 1041386-202312-HR-139-02).

### 2.2 Autonomous wheelchair

**Figure 1:**
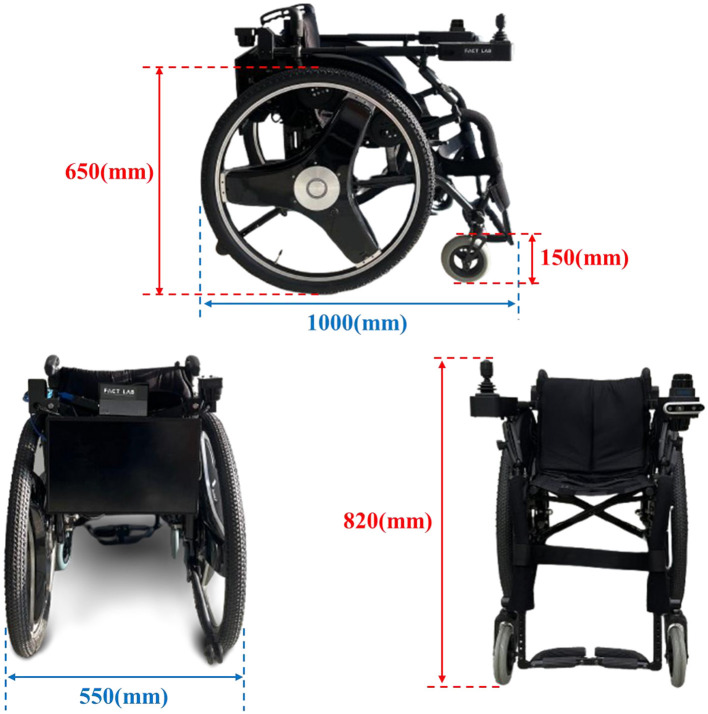
Autonomous wheelchair platform.

For the experiment, an autonomous wheelchair platform equipped with various sensors and a high-performance embedded computer was used (Figure 1). The autonomous wheelchair had overall dimensions of 1,000 mm in length, 550 mm in width, and 820 mm in height. The main wheels had a diameter of 650 mm, while the caster wheels measured 150 mm in diameter. The total weight of the wheelchair was approximately 30 kg, and it was designed to support a maximum user load of up to 100 kg. The wheelchair was powered by a 25 V DC power supply and driven by two 100 W brushless DC (BLDC) motors. User input was provided through a joystick located on the right-hand side, and the system could operate for approximately 3 hours on a fully charged lithium-ion battery. For environmental perception and distance measurement, a LiDAR (Light Detection and Ranging) sensor and a depth camera were mounted on the armrests. In addition, an inertial measurement unit (IMU) was integrated to capture the wheelchair's posture and motion data. All sensor data were processed centrally by a high-performance embedded computer (Jetson AGX Orin, NVIDIA Corporation, Santa Clara, CA, USA), and the wheelchair's real-time position and motion status were displayed on a portable monitor. The autonomous wheelchair employed predefined deceleration and speed control profiles implemented within the onboard control system. Autonomous navigation was performed using a LiDAR-based mapping and localization framework integrated with onboard sensor information for environmental perception and motion control. The predefined deceleration and driving speed modes were implemented by adjusting the wheelchair's braking and motion control parameters within the onboard control system.

### 2.3 Experimental tasks

**Figure 2:**
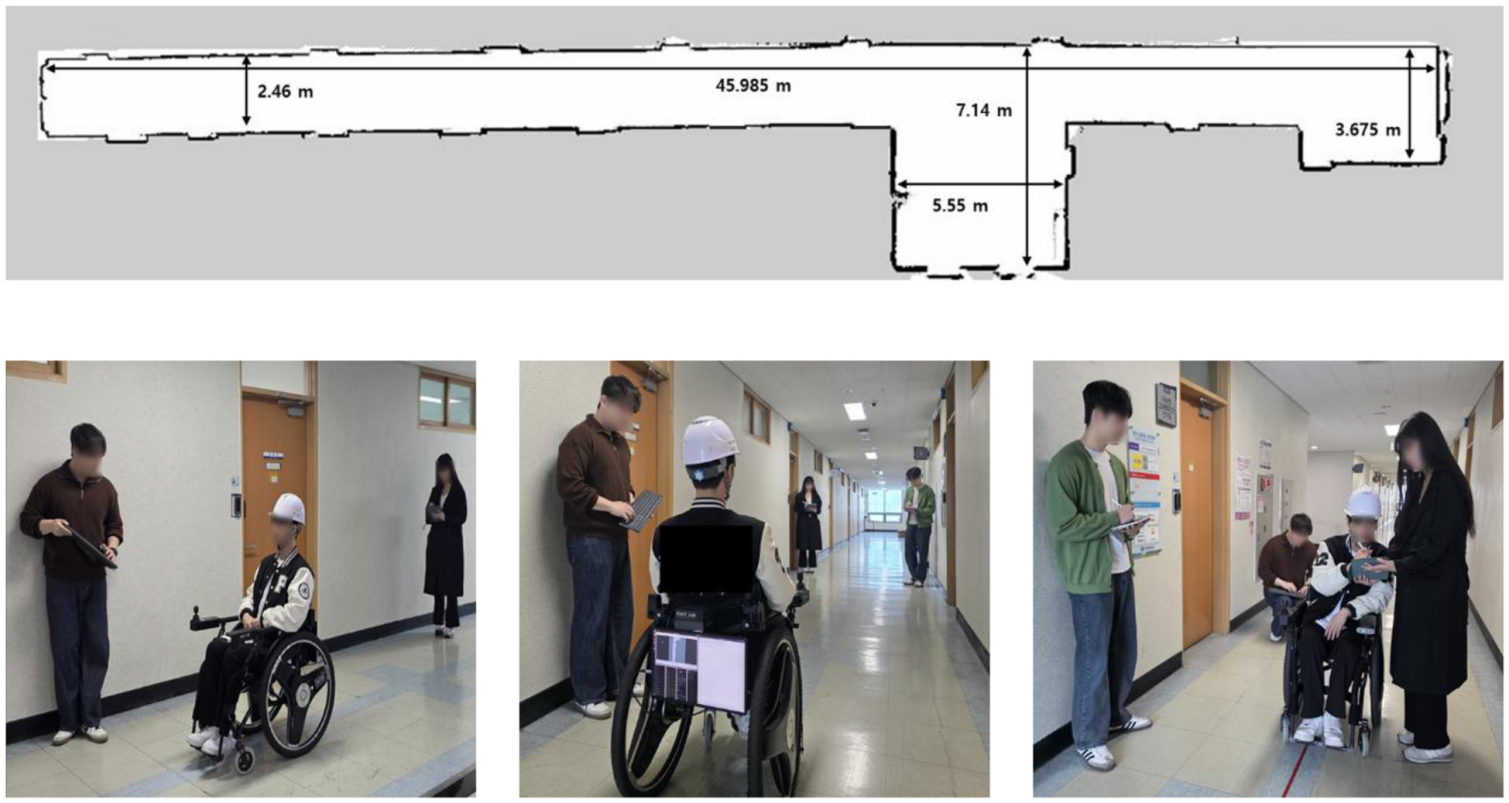
LiDAR point cloud map and experimental environment.

In this study, the experiment was conducted in the corridor on the fifth floor of the Gaon Building at Pukyong National University. The experimental environment, with a total length of approximately 46 m, was designed to support both deceleration driving and driving speed evaluations. Figure 2 presents the LiDAR point cloud map used for the autonomous navigation system, providing additional information regarding the mapping and localization framework employed in the study.

Participants wore safety helmets and conducted the experiment while seated in the autonomous wheelchair (Figure 2). They completed three experimental tasks: (1) a deceleration driving evaluation, (2) a driving speed evaluation, and (3) an evaluation of pre-use and post-use perceptions of the autonomous wheelchair. Both the deceleration driving and driving speed evaluations were conducted using five usability evaluation measures after participants experienced each driving mode. The pre-use and post-use perception evaluation was performed using seven subjective evaluation measures to examine changes in participants' perceptions following use of the autonomous wheelchair. The following sections provide a detailed description of the deceleration driving evaluation, driving speed evaluation, and pre-use and post-use perception evaluation.

#### 2.3.1 Deceleration driving evaluation

**Figure 3:**
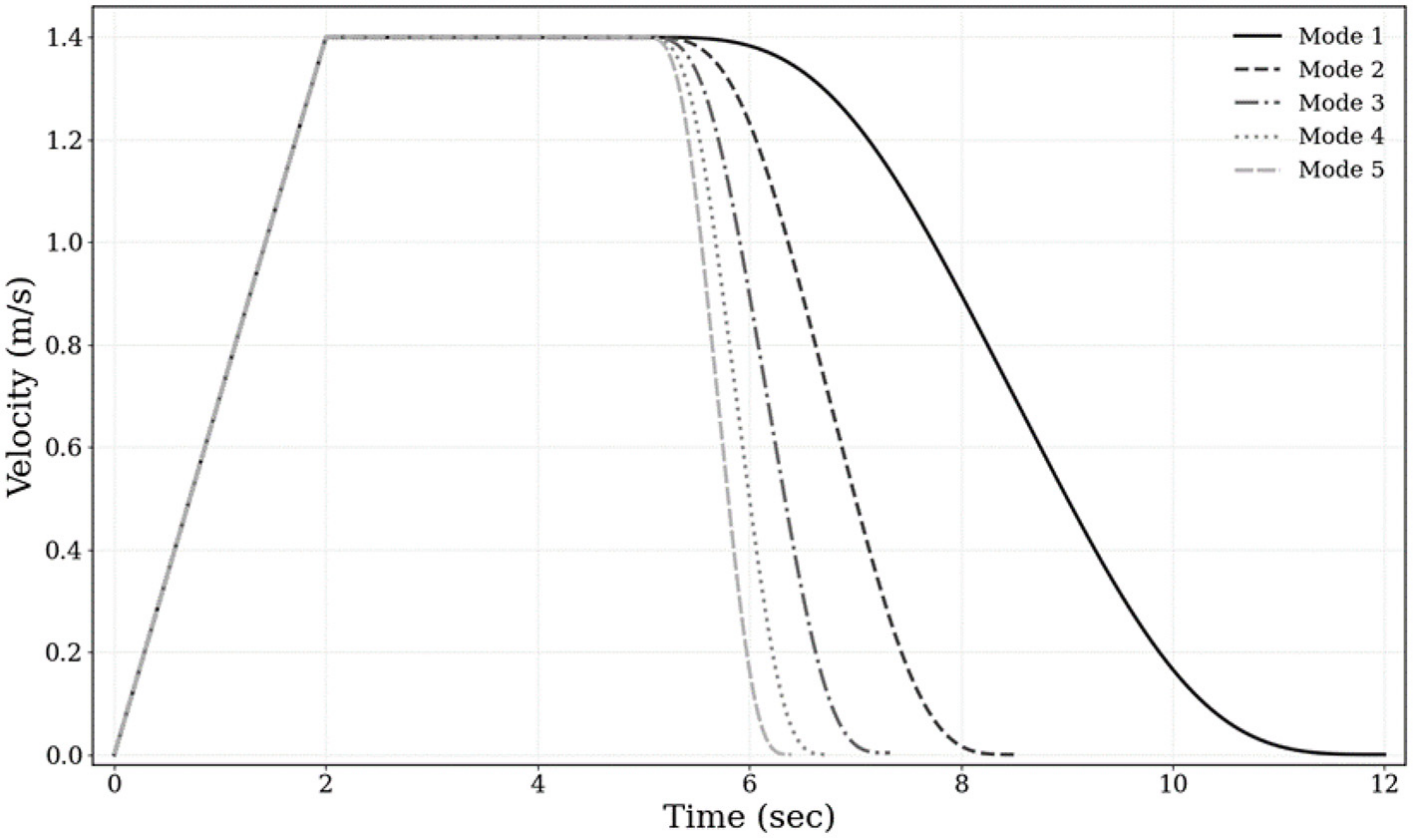
Time-dependent velocity profiles for each deceleration mode.

The deceleration driving evaluation was conducted by having participants board the autonomous wheelchair and experience each deceleration mode. The deceleration modes consisted of five levels: Mode 1 (−0.2 m/s^2^), Mode 2 (−0.4 m/s^2^), Mode 3 (−0.6 m/s^2^), Mode 4 (−0.8 m/s^2^), and Mode 5 (−1.0 m/s^2^). These predefined deceleration profiles were implemented by adjusting the braking control parameters within the onboard control system. The order of the deceleration modes was randomized for each participant. Detailed information regarding the time-dependent velocity profiles for each deceleration mode is provided in Figure 3.

After experiencing each deceleration mode, usability evaluations were conducted using the measures listed in Table 1. All usability evaluations were based on a seven-point Likert scale (1 = strongly disagree, 2 = disagree, 3 = slightly disagree, 4 = neutral, 5 = slightly agree, 6 = agree, and 7 = strongly agree). After completing all deceleration driving evaluations, participants were asked to select their most preferred mode (best mode) and their least preferred mode (worst mode).

**Table 1:** Usability evaluation measures and corresponding descriptions.

Measure	Description
Safety	Decelerating (or driving) the autonomous wheelchair in this mode is safe.
Rapidity	Decelerating (or driving) the autonomous wheelchair in this mode enables quick awareness of the surrounding situation.
Accuracy	Decelerating (or driving) the autonomous wheelchair in this mode enables accurate awareness of the surrounding situation.
Convenience	Decelerating (or driving) the autonomous wheelchair in this mode enhances convenience (comfort and ease).
Preference	I prefer decelerating (or driving) the autonomous wheelchair using this mode.

#### 2.3.2 Driving speed evaluation

**Figure 4:**
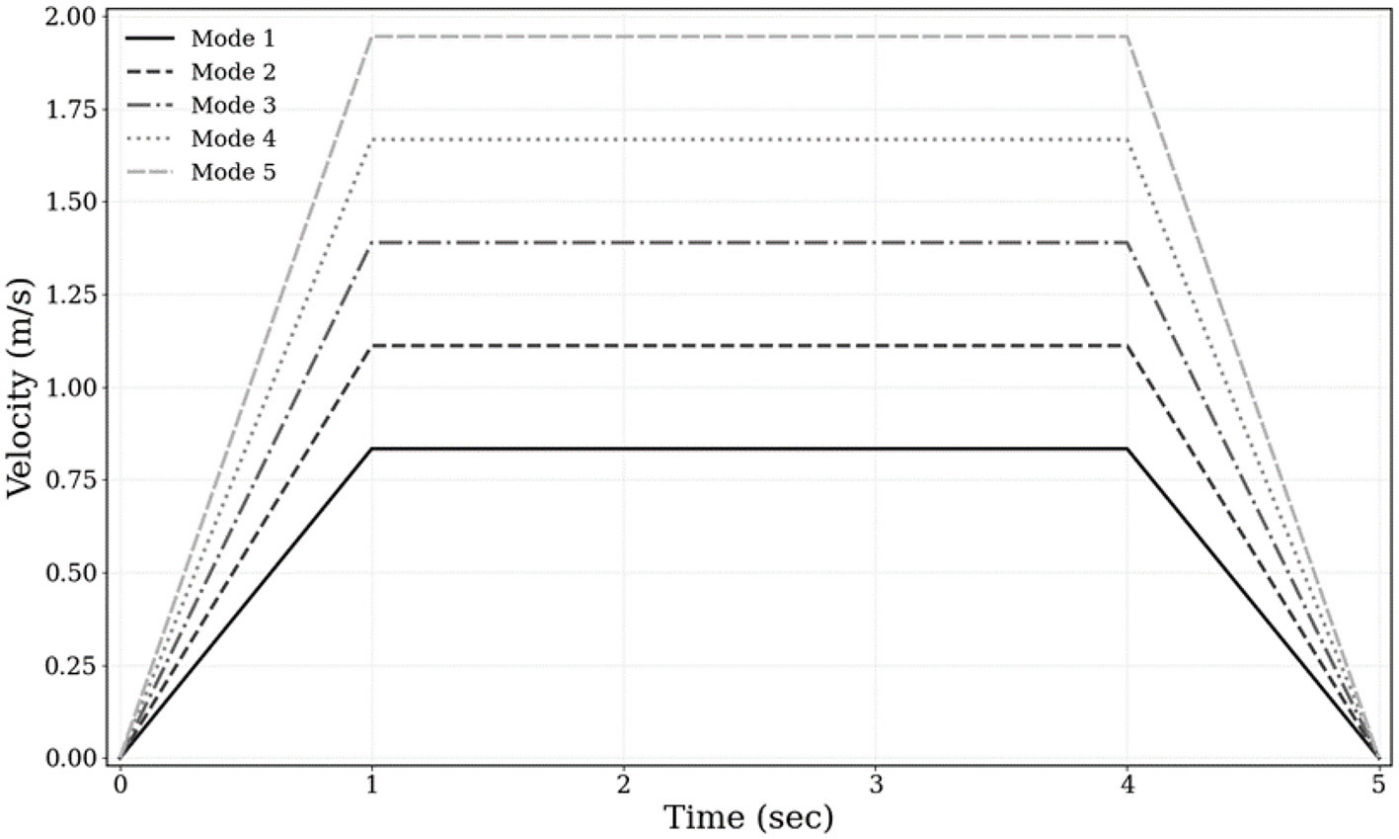
Time-dependent velocity profiles for each driving speed mode.

The driving speed evaluation involved participants boarding the autonomous wheelchair and experiencing each driving speed mode. The driving speed modes consisted of five levels: Mode 1 (3 km/h = 0.83 m/s), Mode 2 (4 km/h = 1.11 m/s), Mode 3 (5 km/h = 1.39 m/s), Mode 4 (6 km/h = 1.67 m/s), and Mode 5 (7 km/h = 1.94 m/s). These predefined driving speed profiles were implemented by adjusting the motion control parameters within the onboard control system. The order of the driving speed modes was randomized for each participant. Detailed information regarding the time-dependent velocity profiles for each driving speed mode is provided in Figure 4.

After experiencing each driving speed mode, participants completed usability evaluations using the measures listed in Table 1. All evaluations were conducted using the same seven-point Likert scale as the deceleration driving evaluation. After completing all driving speed evaluations, participants were asked to identify their most preferred mode (best mode) and their least preferred mode (worst mode).

#### 2.3.3 Evaluation of pre-use and post-use perceptions of the autonomous wheelchair

The evaluation of pre-use and post-use perceptions of the autonomous wheelchair was conducted to examine changes in participants' perceptions following use. Participants completed subjective evaluations both before and after using the autonomous wheelchair, using the measures listed in Table 2. All evaluations were based on a seven-point Likert scale, consistent with that used in the deceleration driving evaluation.

**Table 2:** Perception evaluation measures and corresponding descriptions.

Measure	Description
Intention to use	I am willing to use the autonomous wheelchair equipped with autonomous driving technology.
Reliability	I believe the autonomous wheelchair equipped with autonomous driving technology is reliable.
Safety	I will be able to safely reach my destination using the autonomous wheelchair.
Convenience	The autonomous wheelchair will make the mobility experience more comfortable and enjoyable.
Rapidity	I will be able to quickly reach my destination using the autonomous wheelchair.
Satisfaction	Using the autonomous wheelchair for mobility will enhance satisfaction.
Preference	I prefer using the autonomous wheelchair for mobility.

### 2.4 Experimental procedure

Before the experimental trials, each participant conducted a pre-use perception evaluation of the autonomous wheelchair using the measures listed in Table 2. Participants then underwent a 30-min training session to become familiar with the experimental tasks. Following the training session, the experimental trials—including the deceleration driving and driving speed evaluations—were conducted. During both the training session and the experimental trials, participants wore safety helmets.

For the deceleration driving and driving speed evaluations, each participant first completed five deceleration driving trials (corresponding to the five deceleration modes), followed by five driving speed trials (corresponding to the five driving speed modes). The trial order was randomized for each participant in both evaluations. During each trial, participants experienced one deceleration or driving speed mode, after which usability evaluations were conducted using the measures listed in Table 1. To minimize fatigue, participants were given a minimum rest period of 2 min between trials.

After completing all usability evaluations, participants were asked to identify their most preferred mode (best mode) and least preferred mode (worst mode) for both the deceleration and driving speed conditions. Finally, after all experimental trials were completed, participants conducted a post-use perception evaluation of the autonomous wheelchair using the measures listed in Table 2.

### 2.5 Statistical analyses

The independent variables in this study were deceleration mode (five levels), driving speed mode (five levels), and pre-use and post-use experience with the autonomous wheelchair. The dependent variables included usability evaluation scores (safety, rapidity, accuracy, convenience, and preference) and perception evaluation scores (intention to use, reliability, safety, convenience, rapidity, satisfaction, and preference).

One-way repeated measures analyses of variance (ANOVAs) were conducted to examine the effects of deceleration and driving speed modes on each usability evaluation measure. Mauchly's test was performed within the ANOVAs to assess the assumption of sphericity. In cases where sphericity was not met, the degrees of freedom were adjusted using the Greenhouse–Geisser and Huynh–Feldt corrections (Greenhouse and Geisser, 1959; Huynh and Feldt, 1976). *Post-hoc* tests (pairwise comparisons) were conducted for significant ANOVA results, with the Bonferroni correction applied.

Paired *t*-tests were conducted to compare pre-use and post-use perceptions of the autonomous wheelchair. All statistical analyses were performed using the SPSS version 29.0 (IBM Corp., Armonk, NY, USA), with a significance level (α) of 0.05.

## 3 Results

### 3.1 Deceleration driving evaluation

The ANOVA results indicated that deceleration levels (modes) significantly affected safety, rapidity, convenience, and preference, whereas no significant effect was observed for accuracy (Table 3).

**Table 3:** Usability evaluation results by deceleration mode.

Measure	Mode 1	Mode 2	Mode 3	Mode 4	Mode 5	*p*-value	$\eta_{p}^{2}$
	Mean ±SD	Mean ±SD	Mean ±SD	Mean ±SD	Mean ±SD		
Safety	5.55 ± 1.47	5.95 ± 1.10	5.50 ± 1.19	3.90 ± 1.41	4.10 ± 1.25	**< 0.001** ^***^	0.386
Rapidity	3.85 ± 1.93	5.10 ± 1.33	5.10 ± 1.02	4.40 ± 1.50	5.25 ± 1.29	**0.033** ^*^	0.161
Accuracy	4.20 ± 2.07	5.35 ± 1.42	5.30 ± 1.08	4.55 ± 1.23	5.05 ± 1.36	0.097	0.117
Convenience	5.25 ± 1.83	5.95 ± 1.36	5.55 ± 1.79	4.10 ± 1.59	4.20 ± 1.40	**0.001** ^**^	0.258
Preference	4.70 ± 1.83	5.85 ± 1.36	5.35 ± 1.79	3.70 ± 1.59	3.75 ± 1.40	**< 0.001** ^***^	0.326

^*^p < 0.05, ^**^p < 0.01, ^***^p < 0.001.

**Figure 5:**
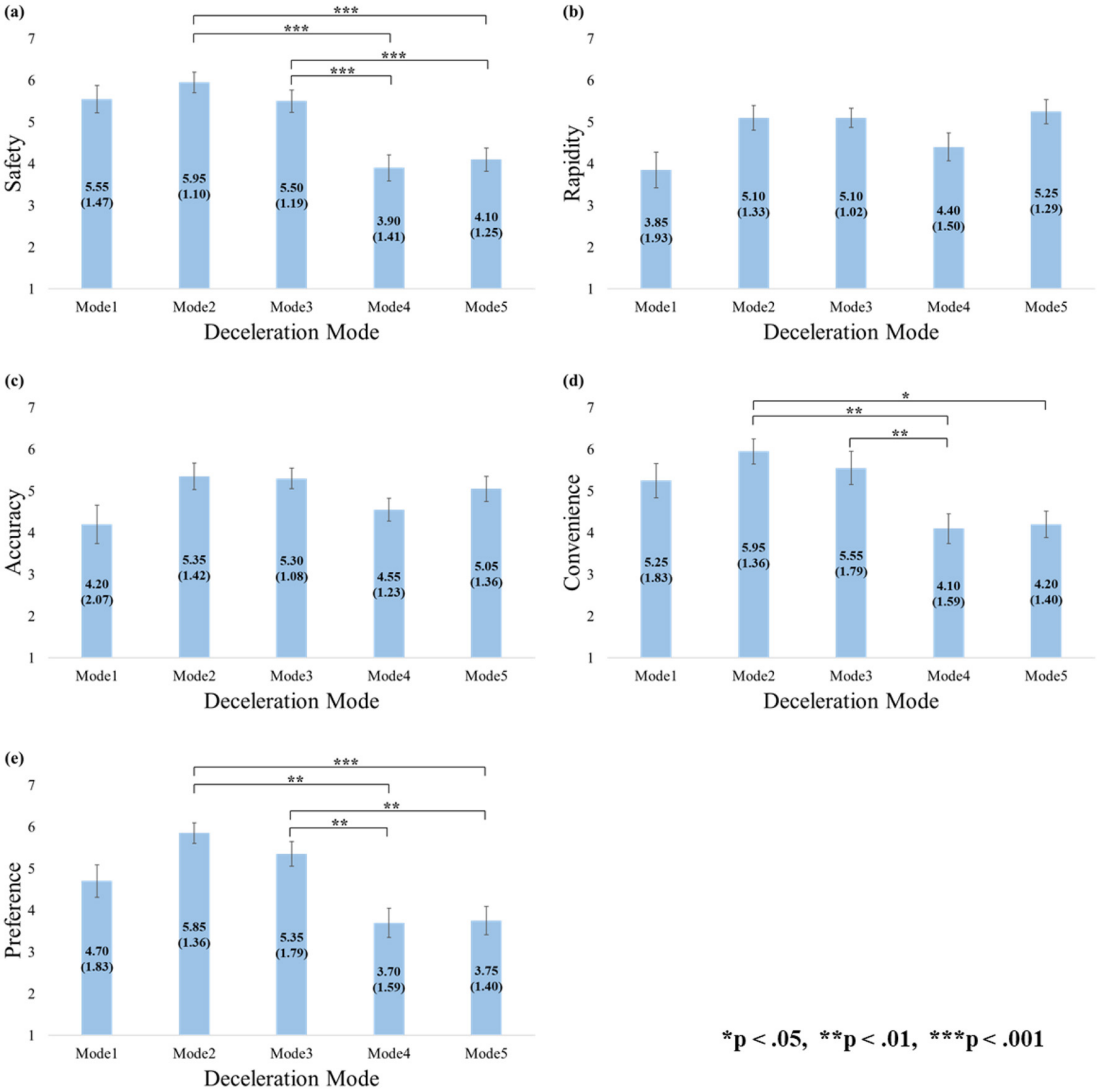
Effects of deceleration mode on usability measures: **(a)** Safety, **(b)** Rapidity, **(c)** Accuracy, **(d)** Convenience, and **(e)** Preference.

For safety and preference (Figures 5a and e), Mode 2 (−0.4 m/s^2^) received the highest ratings, followed by Mode 3 (−0.6 m/s^2^). Both modes showed significant differences relative to Modes 4 (−0.8 m/s^2^) and 5 (−1.0 m/s^2^), which yielded the lowest and second-lowest ratings, respectively. Mean differences, confidence intervals, and *p*-values for the pairwise comparisons were as follows:

- Safety (Mode 2 vs. Mode 4): mean difference = 2.05, 95% CI [0.758, 3.342], *p* < 0.001.
- Safety (Mode 2 vs. Mode 5): mean difference = 1.85, 95% CI [0.739, 2.961], *p* < 0.001.
- Safety (Mode 3 vs. Mode 4): mean difference = 1.60, 95% CI [0.823, 2.377], *p* < 0.001.
- Safety (Mode 3 vs. Mode 5): mean difference = 1.40, 95% CI [0.657, 2.143], *p* < 0.001.
- Preference (Mode 2 vs. Mode 4): mean difference = 2.15, 95% CI [0.517, 3.783], *p* = 0.005.
- Preference (Mode 2 vs. Mode 5): mean difference = 2.10, 95% CI [0.779, 3.421], *p* < 0.001.
- Preference (Mode 3 vs. Mode 4): mean difference = 1.65, 95% CI [0.404, 2.896], *p* = 0.005.
- Preference (Mode 3 vs. Mode 5): mean difference = 1.60, 95% CI [0.331, 2.869], *p* = 0.008.

For rapidity (Figure 5b), Mode 5 (−1.0 m/s^2^) received the highest ratings, whereas Mode 1 (−0.2 m/s^2^) received the lowest ratings. However, the *post-hoc* analysis revealed no statistically significant differences among the deceleration modes.

For accuracy (Figure 5c), although Mode 2 (−0.4 m/s^2^) and Mode 3 (−0.6 m/s^2^) received the highest ratings and Mode 1 (−0.2 m/s^2^) received the lowest, the overall effect of deceleration mode on accuracy did not reach statistical significance (Table 3).

For convenience (Figure 5d), Mode 2 (−0.4 m/s^2^) received the highest ratings, followed by Mode 3 (−0.6 m/s^2^), while Modes 4 (−0.8 m/s^2^) and 5 (−1.0 m/s^2^) yielded the lowest and second-lowest ratings, respectively. Mode 2 exhibited significant differences relative to Modes 4 and 5, whereas Mode 3 showed significant differences relative to Mode 4. Mean differences, confidence intervals, and *p*-values for the pairwise comparisons were as follows:

- Convenience (Mode 2 vs. Mode 4): mean difference = 1.85, 95% CI [0.407, 3.293], *p* = 0.007.
- Convenience (Mode 2 vs. Mode 5): mean difference = 1.75, 95% CI [0.092, 3.408], *p* = 0.034.
- Convenience (Mode 3 vs. Mode 4): mean difference = 1.45, 95% CI [0.311, 2.589], *p* = 0.007.

Following the usability evaluations, participants each selected one best mode and one worst mode for the deceleration condition. Overall, the best and worst mode selections showed a clear pattern favoring moderate deceleration, with the majority of participants selecting Modes 2 or 3 as the best mode and Modes 4 or Modes 5 as the worst mode. The detailed results were as follows.

Best mode selections:

- Mode 2 (−0.4 m/s^2^) was selected most frequently (40.8%, *n* = 8).
- Mode 3 (−0.6 m/s^2^) was selected second most frequently (35.0%, *n* = 7).
- Mode 1 (−0.2 m/s^2^) was selected by 15.0% of participants (*n* = 3).
- Modes 4 (−0.8 m/s^2^) and 5 (−1.0 m/s^2^) were selected least frequently (5.0%, *n* = 1 each).

Worst mode selections:

- Mode 5 (−1.0 m/s^2^) was selected most frequently (55.0%, *n* = 11).
- Mode 4 (−0.8 m/s^2^) was selected by 25.0% of participants (*n* = 5).
- Mode 1 (−0.2 m/s^2^) was selected by 20.0% of participants (*n* = 4).
- Modes 2 (−0.4 m/s^2^) and 3 (−0.6 m/s^2^) were not selected as the worst mode.

### 3.2 Driving speed evaluation

The ANOVA results indicated that driving speed levels (modes) significantly affected all usability evaluation measures (safety, rapidity, accuracy, convenience, and preference) (Table 4).

**Table 4:** Usability evaluation results by driving speed mode.

Measure	Mode 1	Mode 2	Mode 3	Mode 4	Mode 5	*p*-value	$\eta_{p}^{2}$
	Mean ±SD	Mean ±SD	Mean ±SD	Mean ±SD	Mean ±SD		
Safety	6.40 ± 0.75	6.35 ± 0.75	4.70 ± 1.03	3.85 ± 1.31	1.90 ± 0.85	**< 0.001** ^***^	0.818
Rapidity	5.85 ± 1.42	5.75 ± 1.45	5.10 ± 0.79	3.80 ± 1.24	2.40 ± 1.14	**< 0.001** ^***^	0.607
Accuracy	6.00 ± 1.38	5.90 ± 1.21	4.95 ± 0.89	3.55 ± 1.32	2.40 ± 1.10	**< 0.001** ^***^	0.660
Convenience	4.35 ± 1.38	4.60 ± 1.31	5.70 ± 0.73	5.10 ± 1.25	3.70 ± 1.49	**< 0.001** ^***^	0.279
Preference	3.90 ± 1.71	4.35 ± 1.60	5.30 ± 0.98	4.10 ± 1.29	2.60 ± 1.27	**< 0.001** ^***^	0.356

^***^p < 0.001.

**Figure 6:**
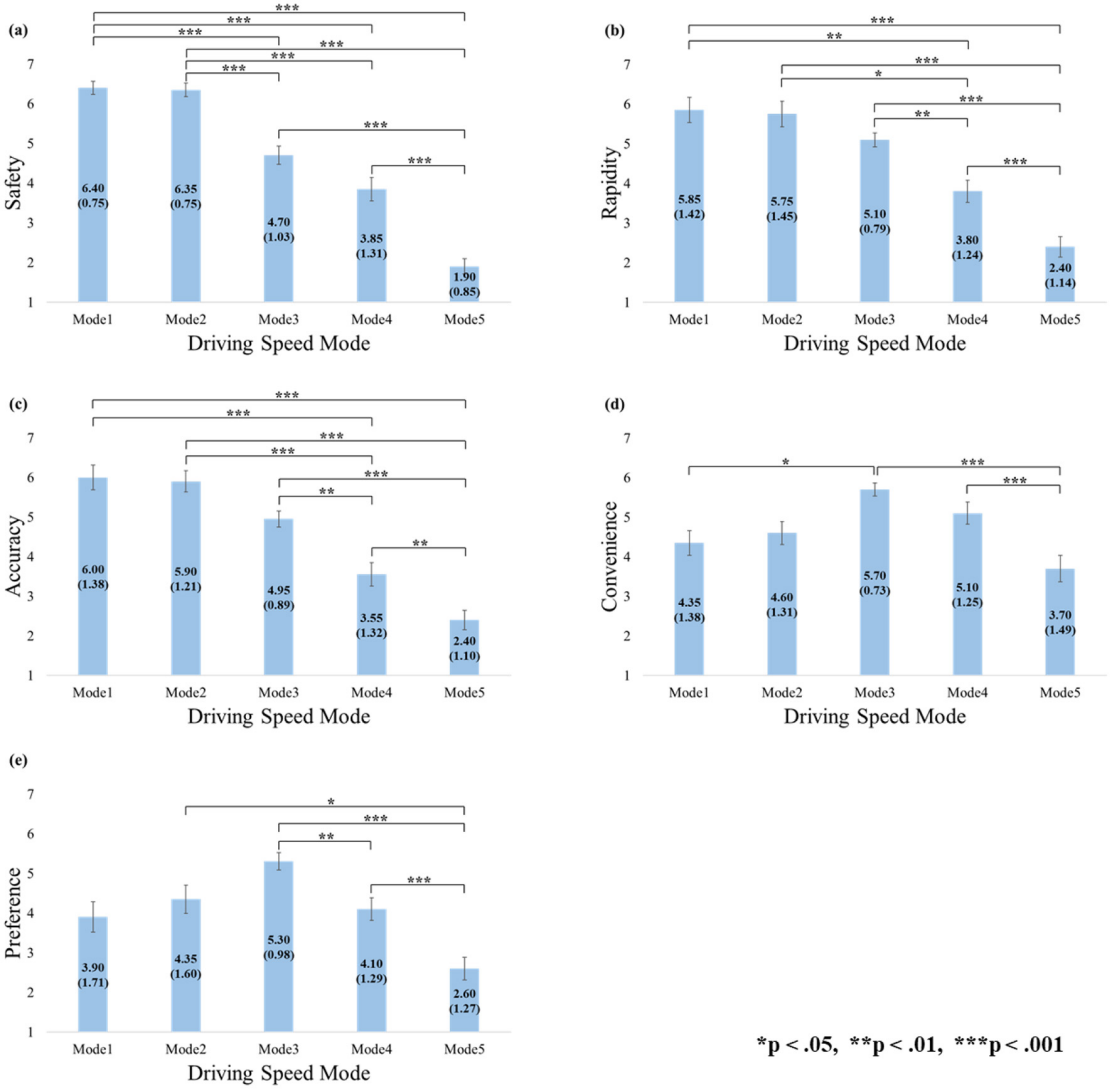
Effects of driving speed mode on usability measures: **(a)** Safety, **(b)** Rapidity, **(c)** Accuracy, **(d)** Convenience, and **(e)** Preference.

For safety, rapidity, and accuracy (Figures 6a–c), Mode 1 (3 km/h = 0.83 m/s) received the highest ratings, followed by Mode 2 (4 km/h = 1.11 m/s). Both modes showed significant differences relative to Modes 4 (6 km/h = 1.67 m/s) and 5 (7 km/h = 1.94 m/s), which yielded the second-lowest and lowest ratings, respectively. Mean differences, confidence intervals, and *p*-values for the pairwise comparisons were as follows:

- Safety (Mode 1 vs. Mode 4): mean difference = 2.55, 95% CI [1.411, 3.689], *p* < 0.001.
- Safety (Mode 1 vs. Mode 5): mean difference = 4.50, 95% CI [3.719, 5.281], *p* < 0.001.
- Safety (Mode 2 vs. Mode 4): mean difference = 2.50, 95% CI [1.315, 3.685], *p* < 0.001.
- Safety (Mode 2 vs. Mode 5): mean difference = 4.45, 95% CI [3.741, 5.159], *p* < 0.001.
- Rapidity (Mode 1 vs. Mode 4): mean difference = 2.05, 95% CI [0.464, 3.636], *p* = 0.006.
- Rapidity (Mode 1 vs. Mode 5): mean difference = 3.45, 95% CI [2.041, 4.859], *p* < 0.001.
- Rapidity (Mode 2 vs. Mode 4): mean difference = 1.95, 95% CI [0.314, 3.586], *p* = 0.013.
- Rapidity (Mode 2 vs. Mode 5): mean difference = 3.35, 95% CI [1.926, 4.774], *p* < 0.001.
- Accuracy (Mode 1 vs. Mode 4): mean difference = 2.45, 95% CI [0.914, 3.986], *p* < 0.001.
- Accuracy (Mode 1 vs. Mode 5): mean difference = 3.60, 95% CI [2.310, 4.890], *p* < 0.001.
- Accuracy (Mode 2 vs. Mode 4): mean difference = 2.35, 95% CI [0.835, 3.865], *p* < 0.001.
- Accuracy (Mode 2 vs. Mode 5): mean difference = 3.50, 95% CI [2.293, 4.707], *p* < 0.001.

For convenience and preference (Figures 6d and e), Mode 3 (5 km/h = 1.39 m/s) received the highest ratings, whereas Mode 5 (7 km/h = 1.94 m/s) yielded the lowest ratings, with significant differences observed between the two modes. Mean differences, confidence intervals, and *p*-values for the pairwise comparisons were as follows:

- Convenience (Mode 3 vs. Mode 5): mean difference = 2.00, 95% CI [.945, 3.055], *p* < 0.001.
- Preference (Mode 3 vs. Mode 5): mean difference = 2.70, 95% CI [1.867, 3.533], *p* < 0.001.

The best and worst mode selections for the driving speed condition revealed a strong preference for intermediate speed, with Mode 3 (5 km/h) being the most frequently selected best mode and Mode 5 (7 km/h) being the most frequently selected worst mode. The detailed results were as follows.

Best mode selections:

- Mode 3 (5 km/h) was selected most frequently (55.0%, *n* = 11).
- Mode 1 (3 km/h) was selected by 20.0% of participants (*n* = 4).
- Modes 2 (4 km/h) and 4 (6 km/h) were each selected by 10.0% of participants (*n* = 2 each).
- Mode 5 (7 km/h) was selected least frequently (5.0%, *n* = 1).

Worst mode selections:

- Mode 5 (7 km/h) was selected most frequently (75.0%, *n* = 15).
- Mode 1 (3 km/h) was selected by 15.0% of participants (*n* = 3).
- Mode 2 (4 km/h) was selected by 10.0% of participants (*n* = 2).
- Modes 3 (5 km/h) and 4 (6 km/h) were not selected as the worst mode.

### 3.3 Comparison of pre-use and post-use perceptions of the autonomous wheelchair

**Figure 7:**
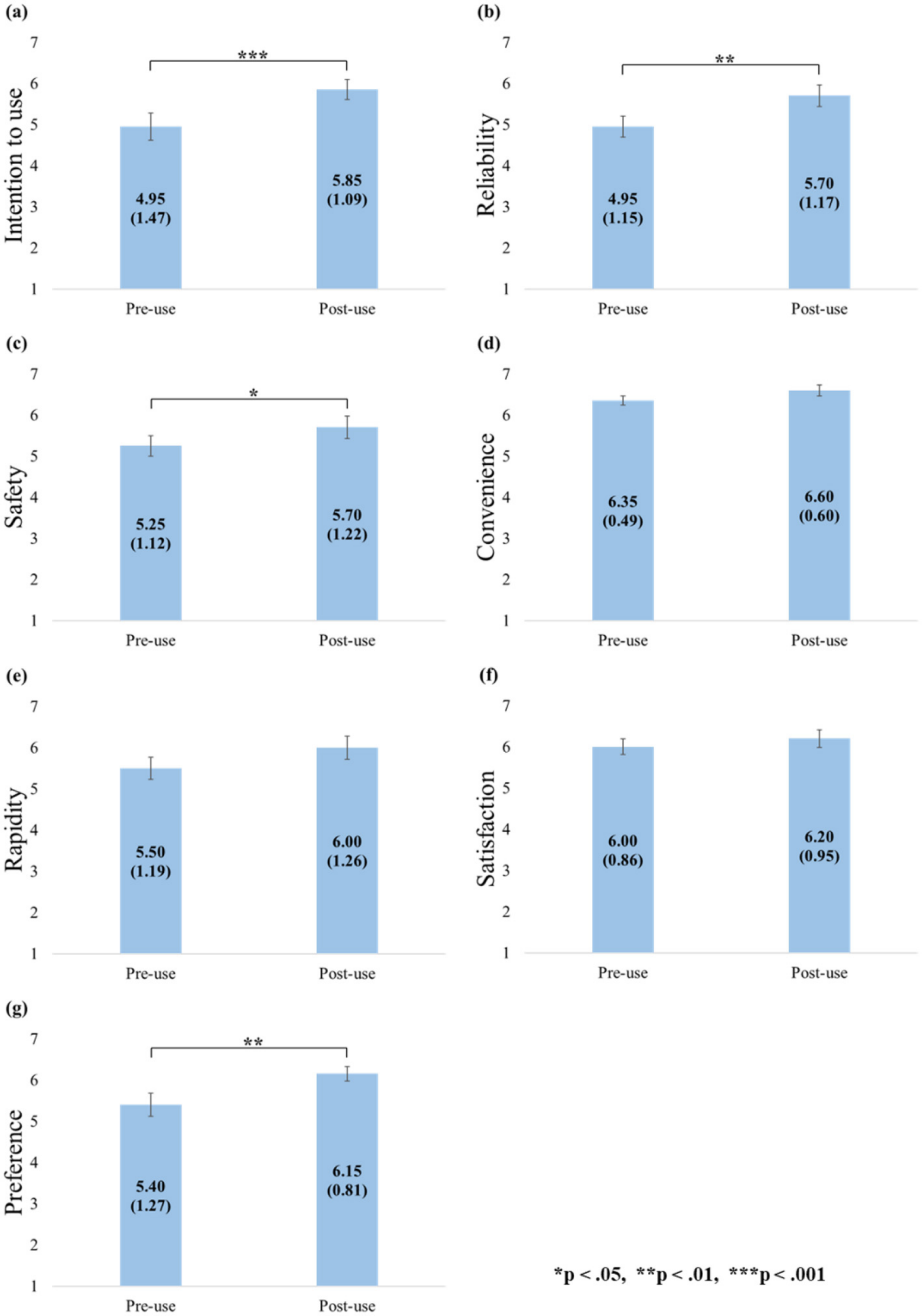
Pre-use and post-use perception ratings for the autonomous wheelchair: **(a)** Intention to use, **(b)** Reliability, **(c)** Safety, **(d)** Convenience, **(e)** Rapidity, **(f)** Satisfaction, and **(g)** Preference.

Paired *t*-tests comparing pre-use and post-use perception ratings revealed significant improvements in four of the seven measures following actual use of the autonomous wheelchair (Figure 7). Specifically, post-use ratings for intention to use (mean difference = 0.90, 95% CI [0.473, 1.327], *p* < 0.001, Cohen's *d* = 0.912), reliability (mean difference = 0.75, 95% CI [0.324, 1.176], *p* = 0.002, Cohen's *d* = 0.910), safety (mean difference = 0.45, 95% CI [0.035, 0.865], *p* = 0.035, Cohen's *d* = 0.887), and preference (mean difference = 0.75, 95% CI [0.298, 1.202], *p* = 0.003, Cohen's *d* = 0.967) were significantly higher than the corresponding pre-use ratings (Figures 7a–c and g), indicating that direct experience with the autonomous wheelchair positively shifted participants' evaluations on these trust-related measures. In contrast, no statistically significant differences between pre-use and post-use ratings were observed for convenience (mean difference = 0.25, 95% CI [−0.085, 0.585], *p* = 0.135, Cohen's *d* = 0.716), rapidity (mean difference = 0.50, 95% CI [−0.015, 1.015], *p* = 0.056, Cohen's *d* = 1.100), and satisfaction (mean difference = 0.20, 95% CI [−0.159, 0.559], *p* = 0.258, Cohen's *d* = 0.768) (Figures 7d–f). These three measures remained relatively stable regardless of whether participants had experienced the system.

## 4 Discussion

This study aimed to investigate the effects of deceleration profiles and driving speed on user experience in autonomous wheelchairs, conceptualized as personal mobility systems. Our findings demonstrate that moderate deceleration profiles (−0.4–−0.6 m/s^2^) received the highest usability ratings in terms of safety, rapidity, convenience, and preference, suggesting that users prioritize a balance between comfort and perceived control. Regarding driving speed, an intermediate speed of 5 km/h showed the highest usability ratings for convenience and preference, whereas lower speeds (3 km/h−4 km/h) were superior in perceived safety, rapidity, and accuracy. Furthermore, direct experience with the autonomous wheelchair significantly enhanced trust-related perceptions, including intention to use, reliability, safety, and preference. These results collectively provide empirical evidence for optimizing the motion planning of autonomous personal mobility devices.

The deceleration mode significantly influenced most usability measures, including safety, rapidity, convenience, and preference, while accuracy remained unaffected (Table 3, Figure 5). Moderate deceleration profiles (−0.4 m/s^2^ and −0.6 m/s^2^) generally received more favorable evaluations than either gentle or aggressive profiles, whereas stronger deceleration modes (−0.8 m/s^2^ and −1.0 m/s^2^) tended to reduce perceived safety, convenience, and overall preference. These patterns were also reflected in the best and worst mode selections, with Mode 2 (−0.4 m/s^2^) and Mode 3 (−0.6 m/s^2^) being most frequently preferred, while Mode 5 (−1.0 m/s^2^) and Mode 4 (−0.8 m/s^2^) were more frequently identified as unfavorable modes.

The preference for moderate deceleration profiles over both overly gentle and aggressive profiles is consistent with previous findings on motion dynamics and wheelchair passenger experience. (Kamper et al. 1999) reported that individuals with spinal cord injuries experienced difficulty maintaining postural stability during sudden deceleration events. Similarly, (Oshima and Momose 2006) demonstrated that abrupt braking in powered wheelchairs can induce upper-body tilt and reduce postural comfort. Consistent with these findings, the present study suggests that aggressive deceleration conditions (−0.8 m/s^2^ and −1.0 m/s^2^) may negatively influence perceived safety, convenience, and overall user preference, even among able-bodied users.

At the same time, the gentlest deceleration condition (Mode 1, −0.2 m/s^2^) did not produce the highest usability ratings across most measures, and 20.0% of participants identified it as the worst mode. This result is consistent with previous findings suggesting that, although gradual deceleration can improve ride smoothness, autonomous mobility systems must still maintain adequate responsiveness to potential hazards in dynamic environments (de Winkel et al., 2023). Excessively slow deceleration may therefore be perceived as insufficiently responsive, potentially reducing perceived safety and system competence. Considering that comfort preferences for deceleration vary across users (Hoberock, 1976), an effective braking strategy should balance smoothness and responsiveness while avoiding both overly gentle and aggressive deceleration.

Beyond the physical aspects of braking, the usability evaluations also appeared to reflect participants' psychological responses to the different deceleration profiles. The finding that safety and preference ratings were highest for moderate deceleration modes, while aggressive modes received the lowest ratings, suggests that participants' evaluations were shaped not only by physical comfort but also by perceived system reliability, competence, and behavioral predictability. Research on automated vehicles has shown that erratic or harsh braking quickly undermines user confidence, whereas smooth and contextually appropriate braking behavior supports perceived safety and trust (Detjen et al., 2021; Koglbauer et al., 2018). (Dettmann et al. 2021) further demonstrated that automated driving styles characterized by higher acceleration and jerk values cause greater subjective discomfort, even when objective vehicle performance remains constant. These findings are consistent with the trust-in-automation framework proposed by (Lee and See 2004), which posits that trust is shaped by the perceived competence and predictability of an automated system. The current results suggest that moderate deceleration profiles (−0.4 m/s^2^−0.6 m/s^2^) effectively communicated system competence to users, whereas aggressive profiles may have been perceived as jerky or unstable, and overly gentle profiles may have appeared hesitant or unresponsive. (Walker et al. 2023) further emphasized that experience-based trust calibration is critical in automated systems, suggesting that deceleration profiles serve as salient behavioral cues through which users evaluate system trustworthiness.

Regarding the effects of driving speed on usability (Table 4, Figure 6), all five usability measures were significantly affected by driving speed. For safety, rapidity, and accuracy, the lowest speeds (Modes 1 and 2, corresponding to 3 km/h and 4 km/h) received the highest ratings, with ratings progressively declining as speed increased. In contrast, for convenience and preference, Mode 3 (5 km/h) received the highest ratings, whereas both the highest and lowest speeds (Mode 5, 7 km/h; Mode 1, 3 km/h) received comparatively lower ratings. These results were corroborated by the best and worst mode selections: Mode 3 (5 km/h) was most frequently selected as the best mode (55.0%), while Mode 5 (7 km/h) was most frequently selected as the worst mode (75.0%).

The finding that lower speeds were associated with higher perceived safety, rapidity, and accuracy is consistent with prior research on the relationship between speed and user experience in autonomous mobility systems. (Petermeijer et al. 2017) reported that higher speeds are associated with increased anxiety and reduced perceived control. The current results extend this finding to the autonomous wheelchair context, demonstrating that speeds beyond 5 km/h progressively diminish users' perceived safety, situational awareness, and navigational accuracy. These results are also in line with the findings of (Wretstrand et al. 2009), who suggested that overall ride conditions, including speed-related factors, contribute to perceived comfort and safety among wheelchair-seated passengers. (Hartwich et al. 2018) similarly reported that driving comfort and acceptance of automated driving are sensitive to the familiarity of driving style, reinforcing the notion that perceived appropriateness of speed is closely tied to the user's expectations and comfort thresholds.

The observation that convenience and preference exhibited a pattern where intermediate speed (Mode 3, 5 km/h) was rated highest, while both lower and higher speeds received lower ratings, suggests the presence of a trade-off between perceived safety and operational efficiency. While lower speeds enhance perceived safety, they may simultaneously be experienced as inefficient or frustrating, reducing overall convenience and preference. Conversely, higher speeds may improve efficiency but undermine comfort and perceived control. This interpretation is consistent with the findings of (Morales et al. 2018), who demonstrated that passenger discomfort emerges from the combined effects of speed and motion dynamics, and with (Shiomi et al. 2015), who found that an autonomous wheelchair adapted to users' preferred speeds while applying gentle braking was rated significantly higher in comfort and acceptance. These results collectively suggest that a driving speed of approximately 5 km/h represents a balanced operating point that optimizes the trade-off between perceived safety and operational efficiency for autonomous wheelchair navigation.

As for the effects of actual use on perceptions of the autonomous wheelchair (Figure 7), post-use ratings for intention to use, reliability, safety, and preference were significantly higher than pre-use ratings, whereas no significant changes were observed for convenience, rapidity, and satisfaction. These results indicate that direct experience with the autonomous wheelchair positively influenced users' trust-related perceptions. The significant increases in intention to use, reliability, safety, and preference suggest that actual interaction with the system enhanced users' confidence in the technology and their willingness to adopt it. This finding is consistent with the observations of (Rushton et al. 2017), who reported that long-term care residents viewed intelligent power wheelchairs as promising tools for improving independent mobility and social participation, even though they also expressed persistent safety concerns. (Xu et al. 2018) similarly reported that direct interaction with autonomous vehicles through field experiments significantly enhanced participants' willingness to adopt the technology, confirming that firsthand experience is a powerful mechanism for trust formation. The current results further suggest that, once users experienced the system firsthand, their safety concerns were substantially alleviated, as reflected in the significant increase in the safety perception rating. This pattern is consistent with (Paddeu et al. 2020), who demonstrated that comfort and trust significantly improved following first-time use of a shared autonomous shuttle vehicle, highlighting the important role of direct experience in shaping user acceptance. (Hoff and Bashir 2015) also noted that prior experience with an automated system is one of the strongest predictors of trust, as it allows users to form accurate mental models of system capabilities and limitations.

The absence of significant changes in convenience, rapidity, and satisfaction between pre-use and post-use perceptions of the autonomous wheelchair (Figure 7) may be attributed to the nature of these constructs, which are more closely related to task-specific performance outcomes than to general trust in the technology. While trust-related perceptions can be substantially influenced by even brief exposure to a functional system, perceptions of convenience, rapidity, and satisfaction may require more sustained use or task-specific optimization to show improvement. This interpretation is supported by the review of (Sahoo and Choudhury 2023), which noted that many smart robotic wheelchairs struggle to achieve sustained real-world adoption, in part due to insufficient attention to user-centered factors such as comfort during everyday use. Similarly, (Beggiato and Krems 2013) demonstrated that user perceptions of convenience and satisfaction in automated driving systems evolve gradually through repeated interactions, rather than being formed during brief initial exposure. Taken together, these findings suggest that sustained engagement over time may be necessary for users to develop stable evaluative judgments regarding task-specific constructs such as convenience and satisfaction.

Overall, the current study empirically investigated the effects of deceleration profiles and driving speed on user experience in autonomous wheelchairs conceptualized as personal mobility systems. The study found that moderate deceleration profiles (−0.4 m/s^2^–0.6 m/s^2^) and an intermediate driving speed (5 km/h) represent an optimal operating point, yielding the highest usability ratings. Furthermore, direct experience with the autonomous wheelchair significantly enhanced trust-related perceptions, including intention to use, reliability, safety, and preference, indicating that user acceptance can be improved through firsthand interaction with the system.

The findings of the current study have practical implications for the design of autonomous personal mobility systems. First, the results suggest that deceleration profiles in the range of −0.4 m/s^2^–−0.6 m/s^2^ should be adopted as a design guideline for autonomous wheelchair braking systems. These moderate deceleration levels were consistently rated highest in safety, rapidity, convenience, and preference, indicating that they offer an effective balance between physical stability and perceived system competence. Second, a default driving speed of approximately 5 km/h appears to represent the optimal operating point for autonomous wheelchair navigation in indoor environments, as it maximized user convenience and preference while maintaining acceptable levels of perceived safety. These specific parameter ranges can serve as practical design references for engineers and developers working on personal mobility systems. Third, the significant improvement in trust-related perceptions following actual use suggests that providing prospective users with opportunities to experience autonomous wheelchair systems firsthand may be an effective strategy for promoting user acceptance and adoption of the technology.

Some limitations of the current study, along with suggestions for future research, are outlined here. First, the present study included only 20 able-bodied participants in their 20 s. Although autonomous wheelchairs were conceptualized as personal mobility systems applicable to broader public and shared environments, the generalizability of the present findings to older adults, individuals with physical impairments, and other demographic groups remains limited. In addition, the relatively small sample size may limit the external validity of the findings, although the within-subject experimental design enabled repeated evaluations across all experimental conditions. Future research should include more diverse participant populations to determine whether the optimal deceleration and driving speed parameters identified in this study are consistently applicable across different user groups. Second, the experimental environment in the present study consisted of a straight indoor corridor designed to minimize environmental variability and isolate the effects of the experimental conditions. However, real-world autonomous wheelchair navigation involves more complex scenarios, including turns, obstacles, uneven surfaces, dynamic pedestrian interactions, and outdoor environments. Therefore, future studies should investigate user experience and usability under more ecologically valid navigation conditions. Third, the present study investigated the effects of deceleration and driving speed independently rather than using a full factorial experimental design. This approach was adopted as an initial-stage investigation aimed at identifying baseline preferred ranges for each parameter, as a full factorial design combining all levels of speed and deceleration would substantially increase participant workload across the repeated subjective evaluations. Previous studies (de Winkel et al., 2023; Morales et al., 2018) have suggested that user experience may be influenced by the interaction between driving speed and braking dynamics. Therefore, future research should employ factorial experimental designs to examine the combined effects of these factors and establish more comprehensive design guidelines for autonomous wheelchair systems. Fourth, the present study primarily relied on subjective usability measures, including safety, rapidity, accuracy, convenience, and preference. Although these measures are important for evaluating user-centered usability, the absence of objective metrics, such as physiological responses, motion stability measures, and collision-related indicators, limits the comprehensiveness of the evaluation. Future research should integrate both subjective and objective measures to provide a more comprehensive assessment of autonomous wheelchair usability and safety. Fifth, the present study evaluated predefined deceleration and driving speed modes under controlled experimental settings rather than adaptive real-time control strategies. In real-world autonomous mobility systems, however, user preferences and comfort levels may vary depending on individual characteristics, environmental context, and situational demands. Future studies should therefore investigate adaptive and user-centered control approaches that dynamically adjust motion parameters based on user state, environmental conditions, and interaction feedback. Sixth, the present study did not consider multimodal interaction factors related to individual user states or behaviors. Future studies could therefore explore multimodal interaction approaches, such as physiological signals, user behavior, and assistive input modalities, to further enhance the personalization of autonomous wheelchair motion control. Finally, the pre-use and post-use perception evaluation was based on a single experimental session. Longitudinal studies examining how perceptions evolve over extended periods of use would provide deeper insights into the long-term user acceptance of autonomous wheelchair systems.

## 5 Conclusions

The present study demonstrates that motion control parameters—specifically deceleration profiles and driving speed—play a critical role in shaping usability and user acceptance of autonomous wheelchairs as personal mobility systems. The findings reveal that users do not simply prefer the gentlest or slowest settings; rather, they favor moderate deceleration and intermediate speed that balance perceived safety with operational efficiency. This preference pattern highlights the importance of designing motion planning algorithms that account for not only physical safety but also the psychological dimensions of user experience, including perceived system competence, predictability, and trust. Additionally, the observed improvement in trust-related perceptions following firsthand experience highlights the value of providing prospective users with opportunities to interact with autonomous wheelchair systems prior to adoption. These results offer actionable design parameters—deceleration profiles in the range of −0.4 m/s^2^–0.6 m/s^2^ and a default driving speed of approximately 5 km/h—that can serve as practical references for the development of human-centered autonomous personal mobility systems, and offer specific design guidelines regarding deceleration and driving speed parameters that balance safety with operational efficiency.

## Data Availability

The raw data supporting the conclusions of this article will be made available by the authors, without undue reservation.
